# Impact of *Clostridium difficile* Infection Versus Colonization on Postoperative Outcomes After Oncological Colorectal Surgery: An Observational Single‐Center Study With Propensity Score Analysis

**DOI:** 10.1002/jso.27923

**Published:** 2024-09-30

**Authors:** Wee Liam Ong, Stefan Morarasu, Sorinel Lunca, Romulus Mihaita Pruna, Cristian Ene Roata, Gabriel Mihail Dimofte

**Affiliations:** ^1^ 2nd Department of Surgical Oncology Regional Institute of Oncology (IRO) Iasi Romania; ^2^ Department of Surgery Grigore T Popa University of Medicine and Pharmacy Iasi Romania

**Keywords:** anastomotic leak, *Clostridium difficile*, colorectal surgery, morbidity

## Abstract

**Background:**

There is limited research available concerning the risk anastomotic leakage in the context of *Clostridium difficile* infection (CDI). Herein, we aim to elucidate the correlation between CDI, encompassing both preoperative asymptomatic *C. difficile* carriers (CDC) and postoperative hospital acquired *C. difficile* infections (HA‐CDI), and the occurrence of anastomotic leakage in patients undergoing oncological colorectal surgery.

**Methods:**

This is an observational, single‐center study. Data were sourced from surgical logs between 2018 and 2023, via the hospital's electronic system. Patients were split into three subgroups: CDC, HA‐CDI, and control group (CG). Groups were compared in terms of patient characteristics, morbidity, and mortality via Fisher's exact test and Kruskal–Wallis test. One‐to‐one propensity score matching was performed to reduce selection bias.

**Results:**

A total of 522 patients were analyzed, split into three subgroups: CDC, *n* = 35; HA‐CDI, *n* = 27; CG, *n* = 460. One‐to‐one propensity score matching reduced the CG to 62 patients. Patients in the HA‐CDI group had higher rates of overall morbidity (*p* < 0.0001), higher rates of anastomotic leaks (*p* = 0.002), more surgical site infections (SSI) (*p* = 0.001), and a longer length of stay (26 vs. 11.2 vs. 9.3 days, *p* < 0.001), while patients in the CDC group had comparable rates of complications with the CG.

**Conclusion:**

HA‐CDI is associated with a higher risk of anastomotic leak after oncological colorectal surgery, while asymptomatic CDC do not have higher morbidity and may be operated electively, under standard CD treatment.

## 1 Introduction

Anastomotic leak (AL) remains the uttermost complication surgeons may face after colorectal resections, being a subject of extensive research and intense debate. Despite advancements made in the way we perform anastomoses to adjust for known risk factors such as tension, perfusion, or surgical technique, it remains a frequent event especially after low colorectal anastomoses. Recently, collagenase producing bacterial strains have been suggested to contribute to the disruption of collagen within the anastomosis leading to a higher chance of leakage [1, 2, 3]. Through this mechanism, colonization with *Enterococcus faecalis* has been proven to increase the risk of AL in both preclinical and clinical studies [4, 5, 6].

*Clostridium difficile* (CD) is a well‐documented microorganism known for its production of tissue‐degrading enzymes, such as proteases and collagenases alongside two toxins: A and B. These toxins are recognized for their proinflammatory and cytotoxic properties, causing disruptions in the actin cytoskeleton and compromising tight junctions. This disruption results in increased permeability and fluid accumulation within intestinal epithelial cells. The potent collagen‐lysing activity and cytotoxic effect of these toxins may hinder the healing process of gastrointestinal anastomoses, consequently reducing the initial strength of the anastomosis and leading to anastomotic leakage [7]. Keshava et al. [8] have raised their concern about the potential role of CDI in the occurrence of ALs in a letter to the editor published 17 years ago. Since then, very few studies [7, 9, 10] have shown that indeed there is a relationship between CDI and anastomotic healing, however none have differentiated between patients colonized with CD and patients that have an acute, postoperative, CDI. For this reason, there is no knowledge of the AL risk of CD carriers that undergo oncological colorectal surgery. Herein, we aim to differentiate between preoperative carriers and acute postoperative infections and analyse, in comparison with CD negative patients, the role of CD on ALs, postoperative morbidity, 30‐day mortality, and length of stay in patients that underwent colorectal cancer resections with anastomosis.

## 2 Materials and Methods

### 2.1 Design and Setting

This is a single center, single department, observational comparative study on patients diagnosed with colorectal cancer who underwent surgery at our institution between 2018 and 2023. All patients underwent standard oncological work‐up and management based on multidisciplinary meetings. All patients were treated and followed at our institution. Our routine preoperative bowel prep includes mechanical prep with PEG solution for left sided colon and rectal resections only. We do not perform oral antibiotic bowel preparation, however a single dose of 500 mg iv metronidazole is added to the cefuroxime based preoperative prophylaxis protocol in patients undergoing colorectal resections. All patients were tested for CD on admission on the day before surgery via a rapid stool test (CerTest Biotec). All patients that tested positive for CD (glutamate dehydrogenase [GDH]+ and toxins A and B +/−) were treated according to our local protocol, in line with international guidelines, by isolation, contact precautions, and vancomycin at an oral dose of 125 mg QDS, which can be increased if required. In cases that did not respond to a single antibiotic regimen, metronidazole was added at a dose of 500 mg TDS, iv. Our institution has a protocol for fidaxomicin escalation or fecal microbiota transplantation (FMT) in non‐responders, however none of the included patients required this. Asymptomatic carriers with both GDH and toxins positive were treated with vancomycin for 10 days due to the theoretical increased risk of AL and postoperative CD exacerbation. Surgery was performed without any delay even in positive cases, under treatment for CD. Informed consent was waived by our Institutions Ethics Committee (Registration Number: 106/29 March 2023) as the research did not pose any risk to the patients and data was anonymized.

### 2.2 Inclusion and Exclusion Criteria

**Figure 1:**
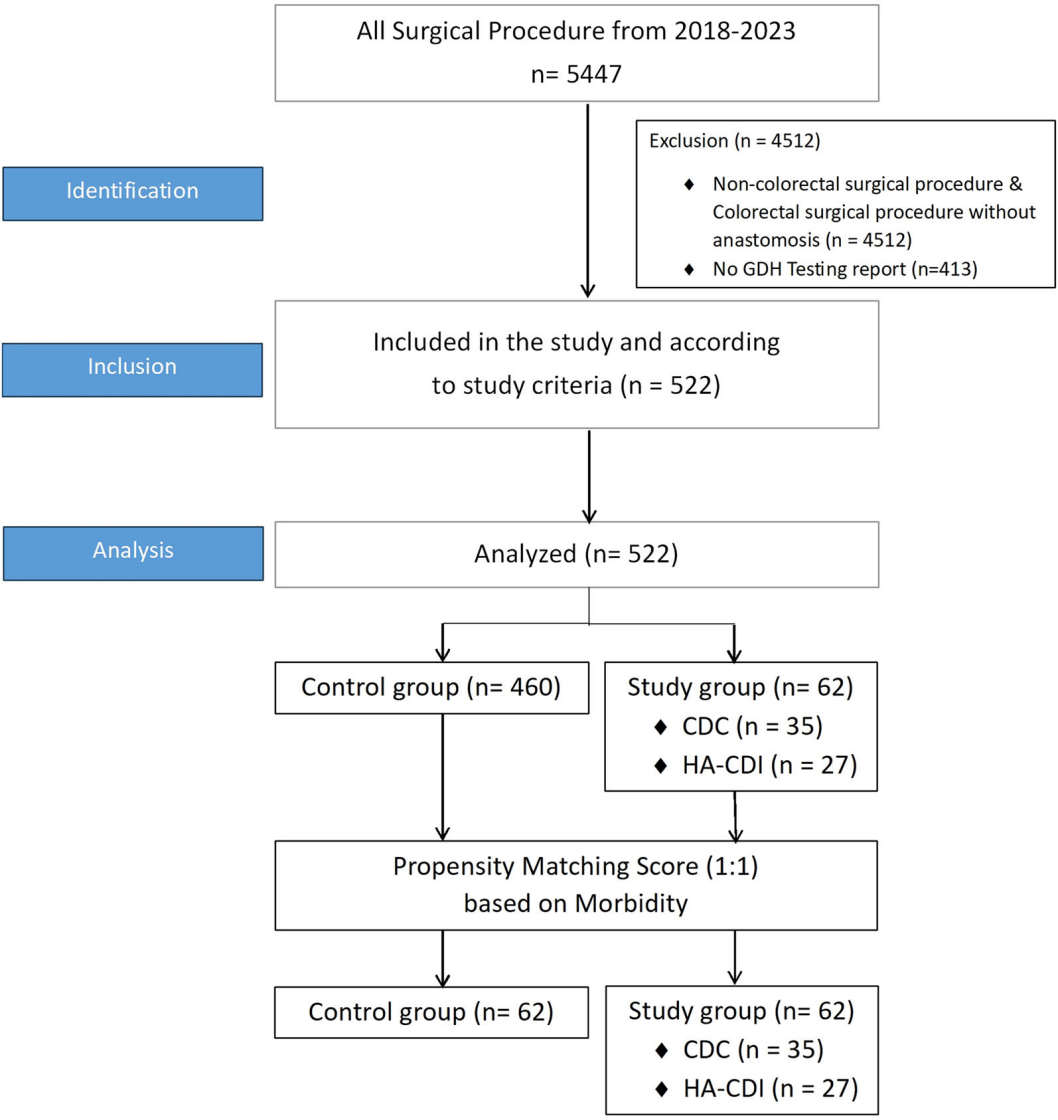
STROBE flowchart.

The STROBE checklist [11] was adhered to (Figure 1). All patients diagnosed with colorectal cancer that underwent resection and anastomosis in our department were included. Patients with abdominoperineal resections, Hartmann's procedures, loop, or end colostomies/ileostomies were excluded. Patients that were not tested for CD preoperatively, were excluded.

### 2.3 Data Analysis

Data were collected from a prospectively maintained database on colorectal resections. Patients were divided into three subgroups: asymptomatic patients that tested positive for CD preoperatively (CDC, *C. difficile* carriers, GDH+, and toxins A and B +/−), patients that became symptomatic and tested positive for CD postoperatively (GDH+ and toxins A and B +/−) after a negative preoperative test (HA‐CDI, postoperative hospital acquired *C. difficile* infections), and patients that tested negative preoperatively and remained asymptomatic or have been tested negative postoperatively (CG, control group). Data on demographics, comorbidities, diagnosis, type of operation, duration of surgery, type of anastomosis, intraoperative complications, pre‐ and postoperative bloods, general, medical and surgical morbidity, in hospital, and 30‐day mortality was extracted. Fisher's exact test for qualitative variables was used instead of *χ* ^2^ test given the low number data set and theoretical counts less than five in some comparisons. Kruskal–Wallis test was used for quantitative variables. The post hoc Dunn's test using a Bonferroni corrected *α* was used to compare mean ranks between the three groups. *p* < 0.05 (95% CI) was considered significant. To further reduce bias from confounding variables and selection bias, propensity score analysis (PSM) was performed using XLSTAT software. Matching control patients in the CDC and HA‐CDI groups were selected according to propensity scores (PSM), in a 1:1 ratio, determined by Mahalanobis distances, with patients in the CG group based on age, comorbidities, anastomosis, type of anastomosis, and preoperative bloods, including preoperative creatinine and albumin levels.

## 3 Results

### 3.1 Cohort Characteristics

Between 2018 and 2023, a total of 935 patients underwent colorectal surgery with anastomosis in our department out of which 522 had preoperative stool test using GDH immunoassays (CerTest Biotec) in addition to molecular testing for toxin gene detection (toxins A and B). The three subgroups consisted of CDC (*n* = 35), HA‐CDI (*n* = 27), and CG (*n* = 460). The average age was 67.02 years and the male‐to‐female ratio was 3:2. The distribution of surgical procedures was as follows: 32.7% (*n* = 171) right hemicolectomies, 0.7% (*n* = 4) patients with left hemicolectomy, 21% (*n* = 110) sigmoid colectomies, 2.1% (*n* = 11) subtotal colectomies, 35.6% (*n* = 186) patients with low anterior resection, and 1.7% (*n* = 9) patients with Hartmann's reversal. In terms of pre‐existing comorbidities, the three subgroups were comparable, except for chronic kidney disease (CKD) which was found to be more frequent in the HA‐CDI group (Table 1), however this difference was balanced after PSM. The type of anastomosis was similar between the three groups, 40.8% (*n* = 213) of anastomoses were performed manually while 59.2% (*n* = 309) were stapled. Preoperative bloods were similar (Table 2).

**Table 1:** Distribution of test results between the two positive subgroups.

	CDC, *n* (%)	HA‐CDI, *n* (%)
Total	35 (100)	27 (100)
GDH+ Toxin A and B+	24 (68.6)	15 (56.6)
GDH+ Toxin A and B–	5 (14.3)	6 (22.2)
GDH+ Toxin A+ Toxin B–	6 (17.1)	6 (22.2)
GDH+ Toxin A– Toxin B+	0 (0.0)	0 (0.0)

*Note:* Results of tests (CerTest Biotec) in the CDC (preoperative) and HA‐CDI (postoperative) groups.

Abbreviations: CDC, *Clostridium difficile* carriers; GDH, glutamate dehydrogenase; HA‐CDI, postoperative hospital acquired *C. difficile* infections.

**Table 2:** Three group comparison in terms of covariates and preoperative characteristics.

	CG, *n* (%)/mean (SD)	CDC, *n* (%)/mean (SD)	HA‐CDI, *n* (%)/mean (SD)	*p* value
Total	460 (100)	35 (100)	27 (100)	
Gender (males)	274 (59.5)	22 (62.8)	18 (66.6)	0.762
Age, mean (SD)	66.7 (10.6)	68.0 (10.3)	70.6 (10.4)	0.123
Diabetes	93 (20.2)	11 (31.4)	5 (18.5)	0.281
IHD	150 (32.6)	16 (45.7)	9 (33.3)	0.292
COPD	21 (4.5)	2 (5.71)	2 (7.4)	0.548
CKD	26 (5.6)	4 (11.4)	6 (22.2)	0.004
PAD	19 (4.1)	0 (0)	1 (3.7)	0.643
Anastomosis				
Ileocolic	163 (35.4)	9 (25.7)	11 (40.7)	0.421
Colocolic	26 (5.6)	2 (5.7)	2 (7.4)	0.814
Colorectal	271 (58.9)	24 (68.5)	13 (48.1)	0.282
Anastomosis type				
Side to side	165 (35.8)	8 (22.8)	12 (44.4)	0.181
Side to end	76 (16.5)	6 (17.1)	1 (3.7)	0.198
End to end	217 (47.1)	21 (60)	14 (51.8)	0.327
Albumin (g/dL)	4.44 (0.4)	4.23 (0.4)	4.58 (0.3)	0.401
Hb (g/dL)	11.9 (2.2)	11.6 (2.2)	11.6 (2.5)	0.342
CRP (mg/dL)	14.8 (25.3)	12.7 (30.5)	16.4 (5.0)	0.419
Creatinine (mg/dL)	0.96 (0.3)	0.97 (0.4)	1.01 (0.3)	0.917
Na+ (mEq/L)	139.6 (2.8)	138.8 (2.6)	139.2 (3.8)	0.836
K+ (mEq/L)	4.2 (0.4)	4.2 (0.4)	4.3 (0.3)	0.910

*Note:* Fisher's exact test for qualitative variables was used instead of *χ* ^2^ test given the low number data set and theoretical counts less than five in some comparisons. Kruskal–Wallis test was used for quantitative variables. The post hoc Dunn's test using a Bonferroni corrected *α* was used to compare mean ranks between the three groups. *p* < 0.05 (95% CI) shows a significant difference in the occurrence of events (for Fisher's test) or significant mean variance (for Kruskal–Wallis test) between the three groups.

Abbreviations: CDC, *Clostridium difficile* carriers; CG, control group; CKD, chronic kidney disease; COPD, chronic obstructive pulmonary disease; HA‐CDI, hospital acquired *C. difficile* infection; IHD, ischemic heart disease; PAD, peripheral arterial disease; SD, standard deviation.

### 3.2 Postoperative Outcomes

**Figure 2:**
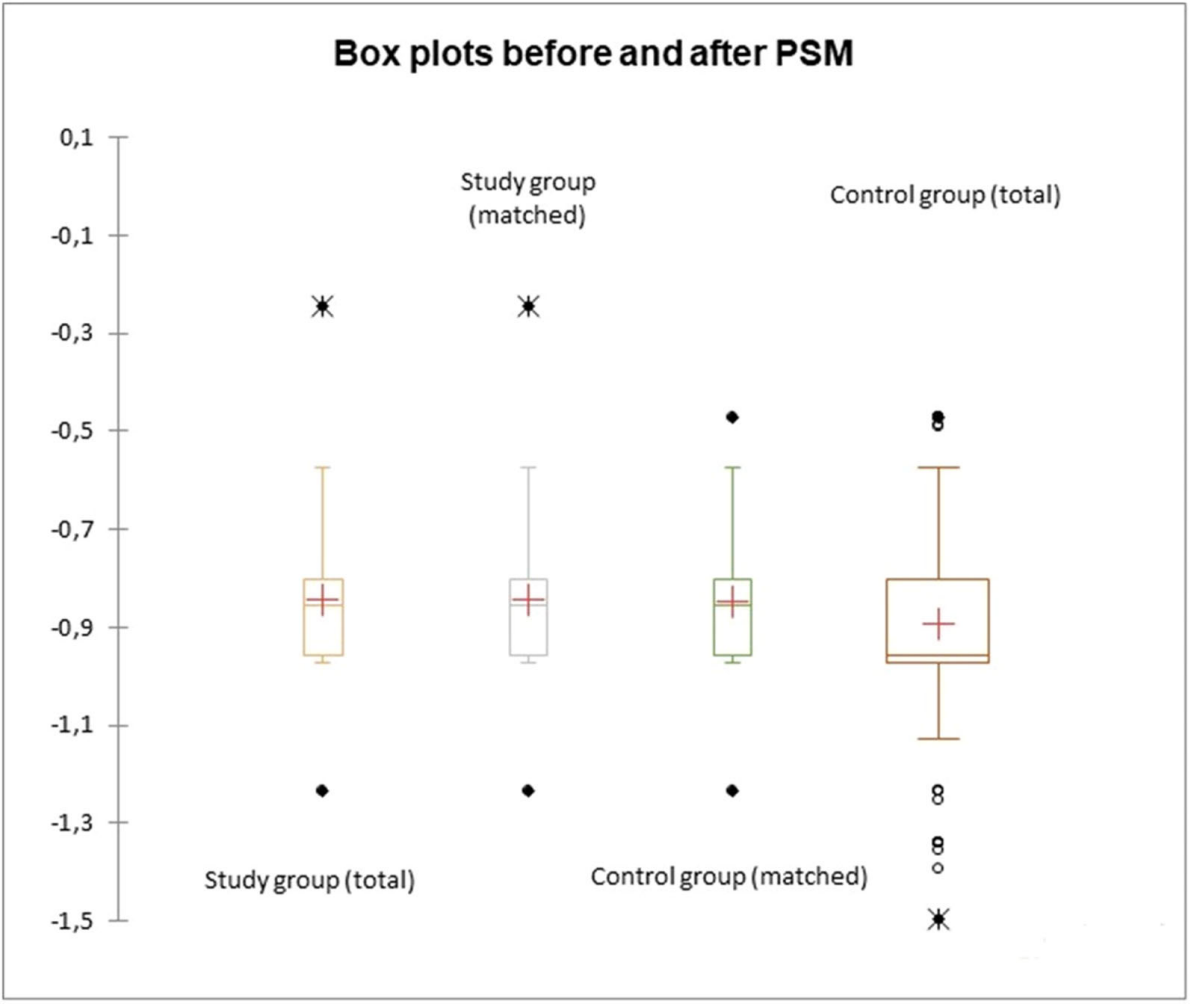
Box plot showing distribution of data before and PSM. Means, range, and outliers are more evenly distributed after PSM based on baseline patient comorbidities.

Table 3 depicts the postoperative outcomes between the three groups. Patients in the HA‐CDI group had higher rates of overall morbidity (*p* < 0.0001), higher rates of ALs (*p* = 0.002), more surgical site infections (SSI) (*p* = 0.001), and a longer length of stay (26 vs. 11.2 vs. 9.3 days, *p *< 0.001), while patients in the CDC group had comparable rates of complications with the CG. Two patients (5.7%) in the CDC group, which were under treatment for CD with po. Vancomycin 125 mg QDS, became CD symptomatic postoperatively and required escalation of treatment with iv metronidazole 500 mg TDS. Postoperative bloods, including creatinine, electrolytes, and albumin level were similar between the three groups. Mortality was also significantly higher in the HA‐CDI compared to the other two. After PSM, groups were evenly distributed (Figure 2) and results remained largely similar, HA‐CDI group having higher rates of morbidity (*p* < 0.0001), ALs (*p* < 0.0001), SSI (*p* = 0.001), and a longer length of stay (*p* < 0.001) (Table 4). After PSM, there was no difference in mortality between the three groups (Table 4). In terms of AL rate, HA‐CDI had a 7.44 higher rate of leak compared to controls (OR: 7.44, 95% CI), whereas CDC had statistically similar rates of leaks compared to controls (OR: 0.76, 95% CI) (Table 5). The HA‐CDI group had a nine times higher rate of morbidity and a five times higher rate of SSI than the CG (Table 5).

**Table 3:** Three group comparison in terms of postoperative outcomes before PSM.

Before PSM				
	CG, *n* (%)/mean (SD)	CDC, *n* (%)/mean (SD)	HA‐CDI, *n* (%)/mean (SD)	*p* value
Total	460 (100)	35 (100)	27 (100)	
Morbidity	80 (17.3)	6 (17.1)	18 (66.6)	< 0.0001
Mortality	4 (0.8)	0 (0)	2 (7.4)	0.048
Anastomotic leak	17 (3.6)	1 (2.8)	6 (22.2)	0.002
SSI	46 (10)	3 (8.5)	10 (37)	0.001
AKI	5 (1.0)	0 (0)	2 (7.4)	0.06
LOS	9.3 (5.5)	11.2 (9.2)	26 (28.0)	< 0.001
Albumin (g/dL)	3.28 (0.58)	3 (0.57)	3.29 (0.66)	0.954
Hb (g/dL)	10.8 (1.6)	10.6 (1.8)	10.6 (2.2)	0.422
CRP (mg/dL)	97.1 (73.6)	102.6 (63.1)	98.2 (82.2)	0.366
NLR	7.33 (4.6)	7.05 (4.8)	9.89 (4.7)	0.889
Creatinine (mg/dL)	0.96 (0.32)	0.94 (0.49)	1.19 (0.48)	0.735
Na+ (mEq/L)	138.3 (2.8)	137.6 (3.3)	137.3 (3.0)	0.901
K+ (mEq/L)	3.95 (0.4)	4.08 (0.5)	3.76 (0.45)	0.925

*Note:* Fisher exact test for qualitative variables was used instead of *χ* ^2^ test given the low number data set and theoretical counts less than five in some comparisons. Kruskal‐Wallis test was used for quantitative variables. The post hoc Dunn's test using a Bonferroni corrected *α* was used to compare mean ranks between the three groups. *p* < 0.05 (95% CI) shows a significant difference in the occurrence of events (for Fisher test) or significant mean variance (for Kruskal–Wallis test) between the three groups.

Abbreviations: AKI, acute kidney injury; CDC, *Clostridium difficile* carriers; CG, control group; HA‐CDI, hospital acquired *C. difficile* infection; LOS, length of stay; PSM, propensity score matching; SD, standard deviation; SSI, surgical site infection.

**Table 4:** Three group comparison in terms of postoperative outcomes after PSM.

After PSM				
	CG, *n* (%)/mean (SD)	CDC, *n* (%)/mean (SD)	HA‐CDI, *n* (%)/mean (SD)	*p* value
Total	62 (100)	35 (100)	27 (100)	
Morbidity	9 (14.5)	6 (17.1)	18 (66.6)	< 0.0001
Mortality	2 (3.2)	0 (0)	2 (7.4)	0.252
Anastomotic leak	0 (0)	1 (2.8)	6 (22.2)	< 0.0001
SSI	4 (6.4)	3 (8.5)	10 (37)	0.001
AKI	2 (3.2)	0 (0)	2 (7.4)	0.252
LOS	8 (3.7)	11.2 (9.2)	26 (28.0)	< 0.001
Albumin (g/dL)	3.24 (0.64)	3 (0.57)	3.29 (0.66)	0.652
Hb (g/dL)	11.1 (1.8)	10.6 (1.8)	10.6 (2.2)	0.545
CRP (mg/dL)	79.6 (47.2)	102.6 (63.1)	98.2 (82.2)	0.131
NLR	6.15 (4.1)	7.05 (4.8)	9.89 (4.7)	0.279
Creatinine (mg/dL)	0.96 (0.33)	0.94 (0.49)	1.19 (0.48)	0.738
Na+ (mEq/L)	138.6 (2.6)	137.6 (3.3)	137.3 (3.0)	0.510
K+ (mEq/L)	3.93 (0.36)	4.08 (0.5)	3.76 (0.45)	0.790

*Note:* Fisher's exact test for qualitative variables was used instead of *χ* ^2^ test given the low number data set and theoretical counts less than five in some comparisons. Kruskal–Wallis test was used for quantitative variables. The post hoc Dunn's test using a Bonferroni corrected *α* was used to compare mean ranks between the three groups. *p* < 0.05 (95% CI) shows a significant difference in the occurrence of events (for Fisher's test) or significant mean variance (for Kruskal–Wallis test) between the three groups.

Abbreviations: AKI, acute kidney injury; CDC, *Clostridium difficile* carriers; CG, control group; HA‐CDI, hospital acquired *C. difficile* infection; LOS, length of stay; PSM, propensity score matching; SD, standard deviation; SSI, surgical site infection.

**Table 5:** OR for postoperative outcomes before and after PSM.

Before PSM	OR	95% confidence limit	*Z* statistic	*p* value
CDC				
Morbidity	0.9828	0.3950–2.4452	0.037	0.9702
Mortality	1.4288	0.0754–27.0725	0.238	0.8121
Anastomotic leak	0.7664	0.0990–5.9344	0.255	0.7989
SSI	0.8438	0.2486–2.8638	0.272	0.7852
HA‐CDI				
Morbidity	9.5000	4.1189–21.9111	5.280	< 0.0001
Mortality	9.1200	1.5936–52.1942	2.484	0.0130
Anastomotic leak	7.4454	2.6620–20.8241	3.826	0.0001
SSI	5.2941	2.2892–12.2436	3.896	0.0001

After PSM				
CDC				
Morbidity	1.2184	0.3944–3.7636	0.343	0.7314
Mortality	0.3408	0.0159–7.3026	0.688	0.4912
Anastomotic leak	5.4348	0.2155–137.0533	1.028	0.3039
SSI	1.3594	0.2862–6.4558	0.386	0.6993
HA‐CDI				
Morbidity	11.7778	4.0499–34.2516	4.528	< 0.0001
Mortality	2.4000	0.3200–17.9976	0.852	0.3944
Anastomotic leak	37.7907	2.0424–699.2446	2.440	0.0147
SSI	8.5294	2.3730–30.6577	3.284	0.0010

Abbreviations: CDC, *Clostridium difficile* carriers; HA‐CDI, hospital acquired *C. difficile* infection; OR, odds ratio; PSM, propensity score matching; SSI, surgical site infection.

## 4 Discussion

This study shows an increased rate of morbidity, ALs, wound infections, and length of stay in patients with acute, postoperative CDI undergoing oncological colorectal surgery, whereas patients who test positive, and are asymptomatic may undergo elective colorectal surgery without additional risks, their morbidity, mortality, and length of stay being similar to patients who are CD negative. To our knowledge, this is one of the few studies to analyse postoperative outcomes in patients with CD and the first one to compare outcomes between CD carriers and patients that develop symptomatic CDI postoperatively. Our study answers a question that may have been raised by surgeons facing patients admitted for elective colorectal cancer surgery which test positive for CD and are asymptomatic. As far as our results show, these patients may be operated electively, with contact precautions, without the need to postpone, considering their oncological diagnosis. At the same time, this research emphasizes the need to be cautious with patients that develop CDI postoperatively. Not only they are at risk of developing AKI, dehydration, and electrolyte disbalances, but they have a seven times higher chance of developing an AL. Their additional morbidity is translated into longer hospitalization and need close follow‐up during their postoperative care.

Collagenase production is one of the key factors in CD gut colonization [12]. Its hydrolytic activity enables bacterial penetration in the intestinal wall facilitating its survival, proliferation, and toxin production. Along with the recent understanding of the role of collagenase producing bacteria in the development of ALs [1, 2, 3, 4, 5], focused research on CD implications in colorectal surgery is deserved, especially considering the high prevalence of CDI, especially in oncological patients, where the incidence of CDI is double compared to the general population [13]. More so, studies showed an increased rate of dysbiosis and CDI incidence after neoadjuvant radiochemotherapy [14] underscoring the importance of CD management in colorectal cancer patients.

Very few studies so far have analyzed the role of CD in AL development, and none have focused on differentiating between asymptomatic carriers and symptomatic positive patients. More so, no study so far analyzed a cancer cohort, where CD colonization is more frequent and may impose delays in cancer therapy due to isolation and treatment of CD. Probably the most notable study on CDI and risk of AL is from Baker et al. [7] which showed an increased leak rate in patients with CDI, from 3.8% to 6.8%. Another study from Calu et al. [9] raised similar concerns, however both studies did not differentiate between preoperative CDC and postoperative CDI. From another perspective, some studies have proven that CD is implicated in pouchitis and pouch failure after proctocolectomy [15, 16]. Interestingly, similar to our results, Lightner et al. [17] showed that preoperative CD colonization (carriers) does not increase the risk of pouchitis or AL. It seems that acute CDI in the postoperative, immunosuppressive state has a greater impact on CD local virulence and subsequent poorer outcomes. Still, this statement warrants further research on the causative factors and pathogenesis of CDI. Other studies on the relationship between CD and colorectal surgery, have shown an increased rate of CDI after ileostomy closure, especially when the closure is delayed [10, 18, 19]. It seems that, CD proliferation and colonization is enhanced in the defunctioned colon. Again, it is difficult to say whether this is related to local dysbiosis, to immunosuppression related adjuvant chemotherapy (reason for delayed closure) or both.

There are limitations to our study. The retrospective nature of the study implies a higher risk of selection bias, although we did try to reduce its impact on the results by performing PSM in a one‐to‐one fashion, extracting comparable patients from the CG. The heterogeneity of the CG was low even before PSM, proven by the overall similar results between the initial groups and matched ones. Another limitation is the relatively small number of patients in the study groups, however the 11.8% incidence of CDI in our cohort is quite high. Considering we focused only on colorectal cancer patients with multiple hospital interactions, it is difficult to believe others will manage to extract higher numbers in a single department, over 5 years. Surely our study cohort will increase in the future and results may be updated as data is still gathered prospectively.

Another source of confounding bias is the fact that carriers were treated with vancomycin due to the increased theoretical risk of AL and CD exacerbation during hospitalization, so we cannot state whether treating CD colonization had an impact on the overall results in the CDC group. Future RCTs could answer this dilemma by comparing outcomes between asymptomatic carriers who receive vancomycin and those who don't. However, the retrospective nature of our study implies a low risk of performance bias (i.e., overtreating carriers to ensure good outcomes). The decision to treat CDC to reduce the risk of active CDI in the postoperative setting was made at the surgeons and infectious disease consultants' discretion and as far as data shows it was an evidence‐based choice considering published literature, including a meta‐analysis showing that CD carriers have a six times higher risk of developing active infection during their hospital stay [20] and this study did not look on cancer patients that undergo colorectal surgery, which are presumably at higher risks. To note, in our study, none of the carriers developed active CDI postoperatively, thus postoperative CD exacerbation may be reduced by treatment of carriers.

We should also consider that CD carriers are not homogenous groups as far as we can easily imagine different levels of colonization in the same category. There is also a limitation in the detection method and detection limit of the method in a rapid immunoassay test as the rapid test (CerTest Biotec) is advertised to have a sensitivity of 96.6% and specificity of 99.4%, but this may be lower, as stated by the producer, in the context of asymptomatic carriers or low colonization levels. The data provided by the producer is on symptomatic CDI. We do not have data on the sensitivity and specificity in asymptomatic carriers, but results from molecular studies [21, 22, 23] demonstrate that bellow threshold CD colonization is between 7% and 26% and may be up to 50% in endemic facilities [22, 23]. In our institution, we use a strict antibiotic prophylaxis regimen with a single dose of cefuroxime and metronidazole during induction of anesthesia, with limited impact on microbiome [24], most probably with little contribution in developing active CDI infection. Whether the addition of metronidazole to the preoperative prep could reduce the risk of postoperative CDI was beyond the scope of our current study, but could be a subject of future research in a case‐control fashion. It is very interesting that the two groups (CG and CDC) behave so similar as it raises the hypothesis that they should be considered and managed in the same way, in the context of colorectal cancer and restrictive antibiotic usage.

## 5 Conclusion

Preoperative CDC do not have a higher rate of morbidity, however acute postoperative CDI increases the rate of AL, morbidity and SSI in colorectal cancer patients undergoing resections and anastomosis, increasing hospital stay. Postoperative CDI should be promptly treated, and patients require a closer follow‐up as they are at higher risk of poorer outcomes, while carriers may be operated electively, with contact precautions and standard CD treatment, without additional risks.

## Ethics Statement

The authors are accountable for all aspects of the work in ensuring that questions related to the accuracy or integrity of any part of the work are appropriately investigated and resolved.

## Conflicts of Interest

The authors declare no conflicts of interest.

## Synopsis

This study is the first to address a dilemma surgeons may face when having to operate a colorectal cancer patient which tested positive for *Clostridium difficile* and is asymptomatic. Our study shows that these patients may be operated electively without the need to postpone. However, patients that develop active infection postoperatively are at a higher risk of anastomotic leak, emphasizing the need to differentiate between preoperative carriers and postoperative symptomatic patients.

## Supporting information

Supporting information.

Supporting information.

## Data Availability

The data that support the findings of this study are available from the corresponding author upon reasonable request.
