# Supplementary material for: Impact of Clostridium difficile Infection Versus Colonization on Postoperative Outcomes After Oncological Colorectal Surgery: An Observational Single‐Center Study With Propensity Score Analysis
Source: J Surg Oncol. 2024 Sep 30;131(3):489–97. doi: 10.1002/jso.27923 (PMC12044277; doi:10.1002/jso.27923)
Supplement: Supplementary file 1 — Supporting information. [file JSO-131-489-s002.pdf]

COLORECTAL DISEASE: Authorship declaration

Title of article: Impact of clostridium difficile infection vs colonization on postoperative outcomes after oncological colorectal surgery: an observational single-centre study with propensity score analysis

PLEASE READ FIRST:
The journal follows the recommendations of the International Committee of Medical Journal Editors (ICMJE) for manuscripts submitted to biomedical journals. According to these, authorship should be based on the following four criteria:

1. Substantial contributions to the conception or design of the work; or the acquisition, analysis, or interpretation of data for the work; AND
2. Drafting the work or revising it critically for important intellectual content; AND
3. Final approval of the version to be published; AND
4. Agreement to be accountable for all aspects of the work in ensuring that questions related to the accuracy or integrity of any part of the work are appropriately investigated and resolved.

All other contributors to the paper should be credited in the 'Acknowledgements' section.

I, the designated corresponding author of the above article submitted for consideration of publication to Colorectal Disease, hereby confirm that:

(i) all named authors agree to the submission of the paper to Colorectal Disease;

(ii) all authors who qualify for authorship under the criteria listed above have been named on the paper;

(iii) the paper is not currently under review by another journal; and

(iv) the paper has not been accepted for publication elsewhere.

Name of corresponding author: Sorinel Luncă

Signature of corresponding author:

Date: 16/07/2024
